# Strategic pheromone signalling by mate searching females of the sexually cannibalistic spider *Argiope bruennichi*

**DOI:** 10.1098/rsos.211806

**Published:** 2022-01-19

**Authors:** Katharina Weiss, Jutta M. Schneider

**Affiliations:** Institute of Zoology, University of Hamburg, Martin-Luther-King Platz 3, D-20146 Hamburg, Germany

**Keywords:** female pheromone, strategic signalling, signalling costs, mate choice, orb-web spider

## Abstract

Reproduction often requires finding a mating partner. To this end, females of many arthropods advertise their presence to searching males via volatile chemical signals. Such pheromones are considered low-cost signals, although this notion is based on little evidence and has recently been challenged. Even when using comparatively low-cost signals, females should signal as little as possible to minimize costs while still ensuring mate attraction. Here, we test the strategic-signalling hypothesis using *Argiope bruennichi*. In this orb-weaving spider, egg maturation commences with adult moult, and females that do not attract a male in time will lay a large batch of unfertilized eggs approximately three weeks after maturation. Using GC-MS analyses, we show that virgin females enhance their signalling effort, i.e. pheromone quantity per unit body mass, with increasing age and approaching oviposition. We further demonstrate that pheromone release is condition dependent, suggesting the occurrence of physiological costs. Mate choice assays revealed that pheromone quantity is the only predictor of female attractiveness for males. In support of the strategic-signalling hypothesis, pheromone signals by female *A. bruennichi* become stronger with increased need as well as body condition, and might thus qualify as an honest signal of female quality.

## 1. Introduction

Searching for a mating partner constitutes one of the costs of sex [1,2]. The costs are particularly high in internally fertilizing gonochoric animals that live solitary, and a high diversity of mechanisms to lure, direct, detect and find partners evolved in this context. Active movement when searching for a partner can entail high costs in terms of energy expenditure [3,4] and predation risk [5–7]. The luring part is usually facilitated by visual, chemical, seismic or acoustic signals and is risky as well [8].

The prevalent sex roles in mate search are signalling females and searching males. A body of theory is concerned with the reasons behind these sex roles, and the most recent theoretical model concludes that female signalling evolves because females tend to benefit less from searching than males [9]. Yet, females still carry costs of signalling, e.g. eavesdropping by predators or parasites, as well as potential production costs. Therefore, it is plausible that selection should favour low-cost signalling modalities. In moths, for example, females generally advertise their presence to searching males with pheromones [10], typically considered to be low-cost signals [11]. However, this notion has been challenged by studies that uncovered considerable physiological costs of pheromone production in different species of moths [12,13]. Overall, the true costs of chemical signalling are still poorly known as empirical tests are scarce [11,14].

An under-appreciated problem for a signalling female is the risk of failing to attract a partner, which is more common than one might assume [15]. Accordingly, selection should produce an optimal balance between the costs and benefits of signalling and favour females that strategically modulate their investment in signalling relative to potential costs such as the perceived risk of mating failure. A recent mathematical model revealed that even small costs can lead to the evolution of strategic adjustments in signalling effort [16]. A meta-analysis of case studies in moths supported the strategic-signalling hypothesis and signified the general pattern that virgin females enhance their signalling effort with age and the associated increased risk of mating failure [16]. However, it is unknown whether these ideas can be generalized since little research exists outside of moths, and we are aware of only one study in Lepidoptera that directly relates enhanced signalling effort to fitness benefits for females [17].

Web-building spiders are another large taxon in which females are found by roving males, and behavioural experiments leave little doubt that female spiders use volatile chemicals to attract males [18–20]. Female web-building spiders are largely stationary whereas adult males are the searching sex, and indeed lose their ability to construct a capture web [21]. Only 11 spider pheromones have been identified thus far [22,23], and there are even fewer quantitative analyses of individual pheromone variation [24,25]. Nevertheless, we argue that spiders make ideal, yet under-appreciated [26], models to study diverse aspects of chemical signalling in mate search, as numerous studies covering a wide taxonomic range show that female spiders' silk potentially conveys fine-tuned chemical information influencing male behaviour. Two patterns are most prevalent. First, males preferentially court or choose virgin rather than mated females (e.g. [27–32]). Second, in some of the above examples, the attractiveness of virgin females was found to increase with age (for a more comprehensive literature review see [23]). As in moths, the latter findings suggest the strategic use of pheromone signals by ageing female spiders.

We present the first experimental test of the strategic-signalling hypothesis in spiders, as we assess quantitative variation in individual female chemical signals within and between age groups and behaviourally test male mate choice using the same females. The European wasp spider, *Argiope bruennichi*, is one of the best-studied spiders concerning sexual biology (e.g. [33]), and it is one of the few spider species in which pheromone chemistry has been investigated [34]. As entelegyne spiders, female *A. bruennichi* have complex reproductive organs with two separate copulatory openings leading to two independent spermathecae [21]. Males use their equally paired pedipalps to transfer sperm but can only inseminate one spermatheca at a time. During copulation, the tip of the sperm-transferring organ breaks off and plugs the mating duct [35,36]. Genital damage and effective mating plugs limit both sexes to a maximum of two copulations. Females are not sperm limited, as a single copulation is sufficient to fertilize several clutches of eggs [37]. Yet, egg maturation commences with adult moult in *A. bruennichi* and proceeds independently of a female's mating status. Unmated females cannot freely delay oviposition and will produce unfertilized egg sacs about three weeks after maturation while remaining receptive. Thus, with every day they remain unmated, females are under increasing pressure to secure at least one mating before the eggs of their first clutch go to waste. This is a considerable fitness cost, as in the laboratory and in the field, females produce only two clutches on average [37]. Moreover, the protandrous males quickly vanish from a population due to sexual cannibalism and their restricted mating rates. Hence, while failure to mate should be infrequent in the high-density patches that are common in this species, late maturing and isolated females are under the risk of experiencing a shortage of males [38].

Female *A. bruennichi* produce the volatile sex pheromone trimethyl (2*R*,3*S*)-methylcitrate (and minor proportions of its (2*S*,3*S*)-stereoisomer; hereafter collectively referred to as trimethyl methylcitrate) [34]. Web silk extracts of virgin adult females, as well as synthetic mixtures of the stereoisomers, have been shown to attract males in the field and elicit courtship behaviour [34]. Importantly, the attractiveness of the pheromone is dose-dependent and higher amounts of synthetic pheromone attract more males [34], although we do not know whether the amounts used in this experiment are within the natural range of pheromone amount. Yet, this shows that females could theoretically augment their chance to attract a male by enhancing the amount of pheromone released.

Behavioural experiments with *A. bruennichi* revealed that males use chemical cues from female body surface and silk to both detect their presence and assess their mating status and body mass [39–41]. Moreover, field observations [42] and laboratory experiments [41] demonstrated that males are more likely to monopolize a female when she is relatively heavy and old, and older females attract more males than young ones in the field [43]. Although male mate choice has been well-studied in several spiders including *A. bruennichi* [20], the female perspective has rarely been looked at. Here, we intend to close this gap by testing the hypothesis that male decisions may be at least partly explained by females strategically modulating pheromone production. As predicted by the strategic-signalling hypothesis, ageing virgin females should intensify pheromone production over time to become more attractive for males.

Capitalizing on a rich knowledge of male mating decisions, we designed standardized bioassays with males as natural receptors of signal intensity [39,40], allowing us to assess the adaptive value of changes in chemical signalling of a female spider. Combining behavioural tests of the males' relative preference for chemical cues of females of different ages and mating status with gas chromatographic-mass spectrometric analyses of pheromone amount, we directly relate signalling effort to female attractiveness.

## 2. Material and methods

### 2.1. Study animals

Subadult *Argiope bruennichi* (Scopoli, 1772) (Araneae, Araneidae) were collected from four different sites in northern Germany (Lower Saxony: Buxtehude, Harmstorf, Pevestorf; Schleswig-Holstein: Wedel) between 24 June and 5 July 2019. *Argiope bruennichi* is common throughout Europe and its collection requires no permits. Spiders were transferred to our laboratory at the University of Hamburg, Germany, where they were individually housed in upturned plastic cups (250 or 500 ml depending on the spider's size) with a hole in the bottom stuffed with cotton wool. Spiders were kept under natural light conditions at a constant temperature of 25°C and a relative humidity of 45%. Twice a week, subadult spiders were provided with approximately 15 *Drosophila* spp. and adult females with three *Calliphora* sp. houseflies. Adult males were fed with approximately 10 *Drosophila* spp. once a week. All spiders were provided with water from a sprayer at least six days a week. Spiders were checked for moults daily and a spider's age is given as days since adult moult. Female size and body mass on the day of the test trial were determined after chemical analysis (see below) to avoid contaminations. Females were weighed on a calibrated scale (Mettler Toledo AB54-S; Mettler Toledo LLC, Columbus, OH, USA) to the nearest 0.1 mg. Mean tibia–patella length of the first pair of legs was used as an index for fixed body size after maturation. Legs were removed from the dead spider, photographed under a stereomicroscope, and measured with the Leica IM500 Imaging software (v. 4.6; Leica Microsystems Imaging Solution Ltd, Cambridge, UK). As an approximation of female body condition, we calculated the residuals of an ordinary least-squares regression of log-transformed body mass at the test day on log-transformed tibia–patella length [44,45]. Since we could not measure tibia–patella length in three individuals, sample sizes differ between analyses.

### 2.2. Behavioural assays

#### 2.2.1. Female test groups

To investigate whether and how females are able to influence their capacity to attract males, we conducted binary choice tests with 120 females that we randomly assigned to four different groups based on their age and mating status in a 2 × 2 factorial design. Females were ‘young' (mean ± s.d.: 6.0 ± 0.8 days, range: 4–7 days) or ‘old’ (mean ± s.d.: 11.8 ± 0.8 days, range: 11–14 days) and ‘virgin' or ‘once-mated'. A previous study had shown that males were only able to distinguish adult from subadult females three days after females' adult moult [40]. Moreover, oviposition normally occurs 15 to 30 days after maturation. Thus, both of our age classes were certainly attractive to males, but differed markedly in the time until the onset of oviposition. Old females were heavier (348.8 ± 101.7 mg versus 197.2 ± 81.3 mg) and consequently had higher body condition (0.11 ± 0.10 versus −0.12 ± 0.11) than young ones due to adult feeding and egg maturation (detailed comparisons of the four female groups are given in table 1 and electronic supplementary material, table S1). Therefore, we did not take body mass or condition into account when assembling female groups.

**Table 1.** Comparison of females of the four groups used in binary choice tests. Values given are means ± s.d. Normally distributed variables (according to Shapiro–Wilk tests) were compared with ANOVA, not-normally distributed variables with Kruskal–Wallis tests (KW). Different superscript letters behind means indicate significant differences between groups based on *post hoc* Tukey's pairwise comparisons for normally distributed variables and Mann–Whitney tests for not-normally distributed variables (detailed test statistics are given in electronic supplementary material, table S1). Numbers in parentheses behind means give the numbers of females (out of 30) for which the variable could be measured/calculated.

	female test group				overall comparisons		
	young virgin	young mated	old virgin	old mated	test	test statistic	*p­*-value
age (d)	6.0 ± 0.8 (*N* = 30)^a^	6.1 ± 0.7 (*N* = 30)^a^	11.8 ± 0.9 (*N* = 30)^b^	11.8 ± 0.9 (*N* = 30)^b^	KW	H = 89.29	<0.0001
body mass (mg)	199.1 ± 79.1 (*N* = 30)^a^	195.2 ± 83.4 (*N* = 30)^a^	334.7 ± 101.0 (*N* = 30)^b^	362.9 ± 100.4 (*N* = 30)^b^	ANOVA	F = 27.02	<0.0001
tibia–patella length (mm)	6.61 ± 0.84 (*N* = 29)	6.92 ± 0.93 (*N* = 28)	6.70 ± 0.95 (*N* = 30)	7.18 ± 0.86 (*N* = 30)	KW	H = 7575	0.06
body condition index	−0.10 ± 0.11 (*N* = 29)^a^	−0.15 ± 0.10 (*N* = 28)^a^	0.13 ± 0.11 (*N* = 30)^b^	0.09 ± 0.10 (*N* = 30)^b^	ANOVA	F = 49.17	<0.0001

To produce once-mated females, virgins were allowed one copulation with a randomly chosen male on the third day after maturation. Matings were observed and males that were not caught by the female after the first copulation were removed from the web to prevent a second mating. This leaves one copulatory opening free for another male and the female technically remains receptive. To ensure comparability between groups, females that caught the male after copulation were prevented from cannibalizing their mate. Once-mated females were subjected to choice tests two days after mating at the earliest to allow for possible chemical changes induced by mating.

#### 2.2.2. Binary choice tests

To assess the preference of naive males (i.e. virgin, without prior contact to females or their silk; mean age ± s.d.: 18.1 ± 1.7 d) for females of the four different groups, we used an established binary choice test set-up consisting of two Perspex frames (35 × 35 × 6 cm), each containing a female in her web. Female *A. bruennichi* rebuild their two-dimensional orb-webs every night and once built, no further silk is added (although holes inflicted by prey might be repaired). Moreover, in the lab, the amount of silk incorporated into a single web is not noticeably influenced by female age or mating status. We generally introduced females into clean frames in the evening and used these webs for testing the next day. We tested all female groups against each other (six different combinations) with ten replicates each (60 trials in total). We did not test females from the same group against each other, however, to keep the number of test trials feasible during the short mating season of *A. bruennichi*. Test pairs were assembled blind with respect to all other female parameters except for age class and mating status. Trials were performed in random order and each spider was tested only once.

The two frames were arranged on a table at an angle of approximately 120°. A silk strand was cut from each web using micro scissors (cutting length 8.5 mm; Carl Roth GmbH, Karlsruhe, Germany) and attached to the tip of an upward facing skewer. The skewer was fixed at an angle of approximately 30° to the table, on which a male was introduced to the set-up with a soft paintbrush (see [31] for a schematic drawing of the set-up). Males tend to walk upward and thus quickly reached the tip of the skewer, often showing typical mate search behaviour, like waving their front legs in the air to detect female pheromone [46]. Upon first contact with the silk strands, males often showed brief bouts of vibratory courtship, before walking up one of the silk stands. A trial was considered valid only if the male had touched both silk strands with its front legs before moving onto one of them. Males were considered as having made a decision when they had walked up at least 2/3 of one silk strand without pausing or turning. Just before entering a web, males were removed to prevent the contamination of the silk, which was subsequently used for chemical analysis (see below). Thus, we cannot say with absolute certainty whether the initial choice of a male would actually have resulted in courtship and mating. However, experience from many earlier mate-choice experiments permits a high confidence in assuming that mating would have followed the decision. After each trial, the skewer was discarded and all instruments were washed with ethanol. An influence of other female signals on male choice is unlikely, although it cannot be ruled out completely. As silk strands were cut from the web and attached to the skewer, their tension was artificially altered, making an influence of vibratory signals or cues unlikely. Moreover, *A. bruennichi* probably has poor eyesight and males were probably not able to judge the females by sight given the distance between frames and skewer.

### 2.3. Chemical analysis

The cuticular chemistry of the females used in the binary choice tests, as well as their web silk was analysed by gas chromatography–mass spectrometry (GC-MS). While only silk was used in the behavioural assays, we also analysed female cuticles to be able to compare pheromone amounts on body and silk. Immediately after each trial, females were removed from their webs and cold anaesthetized at −80°C. Web silk was collected by slowly winding it up on a glass Pasteur-pipette washed in ethanol. The tip of the pipette holding the silk was then snapped off into a small glass vial. All samples were stored at −25°C until analysis. All further processing was done blind regarding female ID and treatment group. Extracts were obtained by placing individual females and silk samples in dichloromethane (DCM; GC-MS grade, Merck KGaA, Darmstadt, Germany) containing known quantities of octadecane (VWR, Darmstadt, Germany; females: 5 µg per sample, silk: 0.5 µg per sample) as an internal standard for quantification of the pheromone. To ensure the proportionality of octadecane and the pheromone trimethyl methylcitrate, calibration curves were created by analysing serial dilutions of synthetic samples of trimethyl citrate (VWR, Darmstadt, Germany), which is chemically very similar to the pheromone, dissolved in DCM containing either 5 or 0.5 µg octadecane each for female and silk samples, respectively. All samples were extracted for 2 h under gentle agitation on a laboratory shaker. Preliminary analyses were conducted to ensure that the long extraction time did not lead to the distortion of pheromone quantification by the extraction of substances from inner tissues. After extraction, female bodies were allowed to dry completely and again stored at −25°C for further measurements.

Due to the generally minute amounts of pheromone, especially in silk samples, extracts had to be concentrated prior to GC-MS analysis. To facilitate and standardize this step, samples were vaporized to dryness at room temperature. Female cuticular samples were redissolved in 90 µl DCM and silk samples were redissolved in 50 µl DCM and transferred to micro-inserts for GC vials. An aliquot of 1 µl of each sample was analysed by a Shimadzu GCMS-QP2010S system (Shimadzu Corporation, Kyoto, Japan), equipped with a SH-Rtx-5MS fused silica capillary column (30 × 0.25 mm ID, 0.25 µm film thickness; Shimadzu Corporation, Kyoto, Japan). The GC was programmed from 80 to 260°C at a constant rate of 30° min^−1^ and from 260 to 300°C at a constant rate of 1° min^−1^, with a 1 min initial isothermal and a 10 min final isothermal hold. A split-splitless injector was operated at a temperature of 250°C in the splitless mode. Carrier gas was helium at a constant flow rate of 1 ml min^−1^. The ionization voltage of the electron ionization mass spectrometer was 70 eV. Source temperature was 200°C and interface temperature was 280°C. Data acquisition and storage were performed with the software GCMSsolution (v. 4.45; Shimadzu Corporation, Kyoto, Japan). Peak areas were obtained by manual integration using the GCMSsolution software.

The previously identified volatile pheromone trimethyl methylcitrate was identified by comparing its characteristic mass spectrum to previously published spectra [34]. We did not differentiate between the two stereoisomers, as their ratio generally varies and there are currently no indications that their ratio influences the attractiveness of the pheromone [34].

### 2.4. Data analysis

#### 2.4.1. Comparison of pheromone amount on female cuticle and silk

We obtained chemical profiles for all 120 females from the binary choice dataset and webs of 114 of these females (no GC-MS data could be obtained for six webs, as the amount of silk was too small). Absolute pheromone amounts were calculated using the internal standard peak. Statistical analyses of pheromone amount were performed with the statistical software package PAST v. 3.25 [47]. Non-parametric tests were used if data could not be transformed to normality and homogeneity of variances could not be obtained (e.g. due to many zero values for pheromone amount because mated females generally and young females often do not release pheromone, see Results). Generally, outliers (larger than 1.5 times the interquartile range between Q1 and Q3) were included in the statistical analyses, while extreme values (larger than three times the interquartile range between Q1 and Q3) were excluded from statistical analyses (therefore, sample sizes may differ, e.g. between calculations of means and statistical tests). To see if pheromone amount is influenced by female condition, ordinary least-squares regressions of log-transformed pheromone amount on the cuticle on body condition were conducted for virgin females of both age groups separately (since body mass is not independent of female age, table 1). A reduced major axis regression was used to test for a correlation of pheromone amounts on cuticles and silk [48]. To test whether females actively increase their signalling effort, we compared pheromone amount per unit body mass between virgin females of both age groups.

#### 2.4.2. Male mate choice

To assess the influence of female variables on male decisions in binary choice tests, we set up a binary logistic regression model in R v. 4.0.0 [49]. We not only wanted to assess which female variables influence male choice, but also how males perceive differences between females, i.e. whether they assess female parameters directly (e.g. by gaining information on a female's size or body mass visually or via web tension) or whether they use chemical cues as proxies. Therefore, we included four variables of female state and condition as well as pheromone amount on web silk into the model. We used chemical data from web silk since in our test set-up males had direct contact to the silk, but not to the female bodies. We had to exclude six of the 60 test trials due to missing values. ‘Choice for left female' was chosen as ‘neutral’ dependent variable so that ‘age class’ and ‘mating status' could be used as independent factors. We added the following explanatory variables to the model (continuous variables were computed as the differences between the left and the right female): (i) body mass difference between females, i.e. relative mass of the left female, (ii) mating status (coded as a dummy variable with four categories: both virgin, both mated, left virgin/right mated, left mated/right virgin), (iii) whether the left female originated from the same site as the male or not, (iv) age class (coded as a dummy variable with four categories: both young, both old, left young/right old, left old/right young), and (v) relative pheromone amount on the web of left females. According to a Shapiro–Wilk test, the variable ‘relative pheromone amount' differed from a normal distribution and transformations did not change this. However, the visual inspection of the untransformed data did not show a large deviation from a normal distribution, and we decided to include it untransformed. We simplified the model by removing one variable at a time, starting with the least significant one [50]. Annotated R-code is provided in the electronic supplementary material. We conducted a binary logistic regression model testing potential influences of male age or collection site, as well as environmental factors (i.e. start time of trial, relative humidity and room temperature during trial) on ‘choice for left female'. None of these parameters influenced male decisions and we excluded them from further analyses.

## 3. Results

### 3.1. Variation in pheromone amount

**Figure 1.**
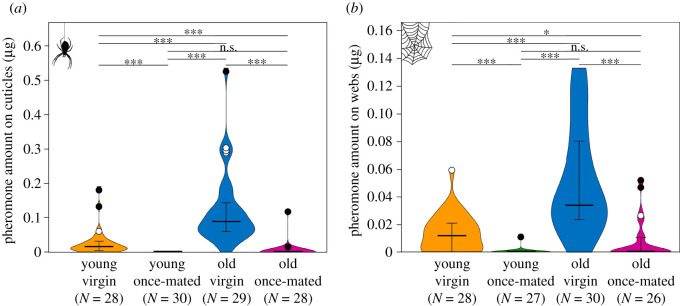
The amount of the volatile pheromone trimethyl methylcitrate clearly differs between female states for (*a*) cuticular and (*b*) web silk samples. Violin curves represent highest and lowest values and distribution of measured values, bold horizontal lines within violins represent medians, whiskers represent 25–75% quartiles, open circles indicate outliers (included in statistical analysis), filled circles indicate extreme values (excluded from statistical analysis). Asterisks indicate significant differences between groups based on Mann–Whitney pairwise comparisons (see text) at the following significance levels: *p < 0.05, ***p < 0.0001; n.s., not significant. For statistical details, see electronic supplementary material, table S2. Numbers in parentheses below each group on the *x*-axis denote sample sizes (without extreme values).

The amount of the volatile pheromone trimethyl methylcitrate was generally much higher on females' cuticles than on web silk, but the quantities were strongly correlated across all female groups (reduced major axis regression: *R* = 0.831, *p* < 0.0001, *N* = 114) and hence were both informative. Only virgin females possessed substantial amounts of pheromone on their cuticle and silk, and old virgins had by far the highest amounts, whereas once-mated females had only small amounts of pheromone regardless of age (figure 1). Kruskal–Wallis tests confirmed the overall statistical significance of these differences for both cuticular (H = 81.35, *p* < 0.0001, *N* = 115) and silk samples (H = 57.89, *p* < 0.0001, *N* = 111; *post hoc* pairwise comparisons are given in electronic supplementary material, table S2).

**Figure 2.**
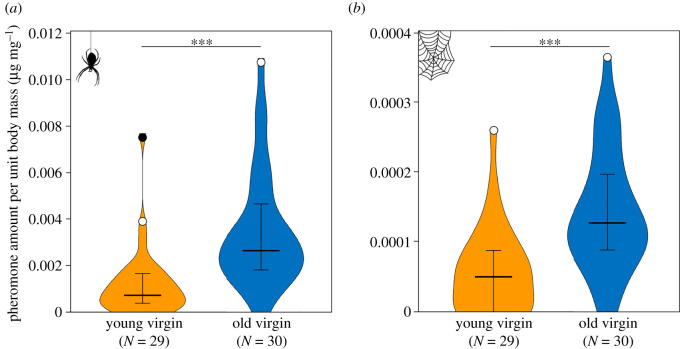
Old virgin females possess higher relative amounts of the volatile pheromone trimethyl methylcitrate per unit body mass than young ones on (*a*) their cuticle and (*b*) their web silk. Violin curves represent highest and lowest values and distribution of measured values, bold horizontal lines within violins represent medians, whiskers represent 25–75% quartiles, open circles indicate outliers (included in statistical analysis), filled circles indicate extreme values (excluded from statistical analysis). Asterisks indicate significant differences at *p* < 0.0001 based on Mann–Whitney comparisons (see text). Numbers in parentheses below each group on the *x*-axis denote sample sizes (without extreme values).

In old virgin females, pheromone amount increased significantly with body condition (ordinary least-squares regression: *R* = 0.48, *p* = 0.007, *N* = 30), while in young females there was a not significant trend in the same direction (*R* = 0.36, *p* = 0.056, *N* = 29). Moreover, old virgins had more pheromone per body mass than young ones on their cuticle (median and interquartile range: old virgin: 0.0027 µg mg^−1^, range: 0.0018–0.0046 µg mg^−1^, *N* = 30; young virgin: 0.0008 µg mg^−1^, range: 0.0004–0.0017 µg mg^−1^, *N* = 30; Mann–Whitney: U = 114, *p* < 0.0001, *N* = 59; figure 2*a*) and their silk (Median and interquartile range: old virgin: 0.0001 µg mg^−1^, range: 0.0001–0.0002 µg mg^−1^, *N* = 30; young virgin: 0.0 µg mg^−1^, range: 0.0–0.0009 µg mg^−1^, *N* = 28; Mann–Whitney: U = 170, *p* < 0.0001, *N* = 58; figure 2*b*).

### 3.2. Male mate choice assays

**Figure 3.**
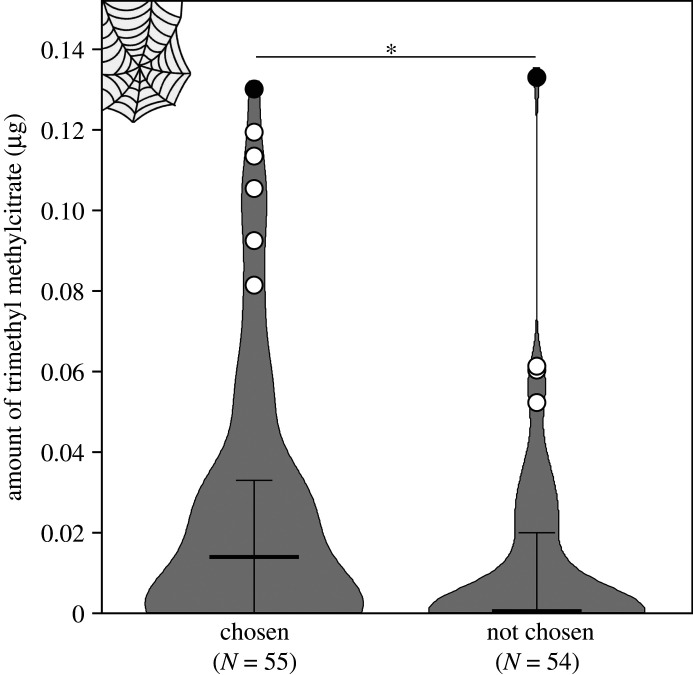
Females that have been chosen by males in the binary-choice tests possess larger amounts of pheromone on their webs than females that have not been chosen. Violin curves represent highest and lowest values and distribution of measured values, bold horizontal lines within violins represent medians, whiskers represent 25–75% quartiles, open circles indicate outliers (included in statistical analysis), filled circles indicate extreme values (excluded from statistical analysis). Asterisk indicates significant difference with *p* = 0.025 based on a Mann–Whitney comparison (see text). Numbers in parentheses below each group on the *x*-axis denote sample sizes (without extreme values).

Before they were subjected to chemical analyses, each female took part in binary choice assays to assess the preference of naive males for silk of females of different states. Thus, we can directly test whether the pronounced differences in pheromone amount influence male decisions. Indeed, females that were chosen by a male possessed significantly more (median: 0.015 µg, interquartile range: 0.0–0.033 µg, *N* = 56) pheromone on their webs than females that were not chosen (median: 0.0 µg, interquartile range: 0.0–0.021 µg, *N* = 58; Mann–Whitney: U = 1255, *p* = 0.025, *N* = 109; figure 3). A binary logistic regression model with ‘choice for left female’ as the dependent variable and two manipulated factors (female relative age and mating status), one control factor (collection site), as well as relative body mass as non-chemical covariate and relative pheromone amount on webs as chemical covariate revealed the difference in pheromone amount on webs as the only significant predictor for male mate choice (*Z* = 2.37, *p* = 0.018, *N* = 54). The result is not influenced by covariances, as a model including all above variable except pheromone quantity yields no significant explanatory factor for male choice. Details of male choice decisions between the different female groups are given in electronic supplementary material, table S3. The time males took to make a decision did not differ between female groups (one-way ANOVA: *F* = 1.268, *p* = 0.291, *N* = 60; electronic supplementary material, table S3).

## 4. Discussion

We demonstrate that female *Argiope bruennichi* tailor pheromone release to mitigate the risk of losing fitness by remaining unmated until the onset of egg-laying. We further demonstrate that increasing pheromone release is adaptive and enhances female mating chances as in binary choice tests males based their mate choice solely on relative pheromone quantity on females' web silk. These findings support the strategic-signalling hypothesis and are in line with model predictions by Umbers *et al*. [16], who used mathematical modelling to compare different signalling strategies based on data from the comparatively well-studied Lepidoptera.

We were able to quantify pheromone amounts on the body and silk of females that were either once-mated (leaving one genital opening free for another mating) or selected to remain virgin for a short (young virgins) or long (old virgins) time after maturation. Our results clearly show that the amount of pheromone they release covaries with reproductive status and age. Only virgin females possessed substantial amounts of pheromone, and those that were kept unmated until they were close to oviposition significantly increased their investment in signalling. The increase was apparent as absolute pheromone amount on body surface and silk, as well as relative to female body mass. As female *A. bruennichi* normally dismantle and rebuild their webs every night, we sampled all analysed webs within one day after their construction. Hence, increased pheromone amount on webs of older females is not due to simple accumulation over time (although this cannot be ruled out for pheromone amount on female cuticle). We thus provide experimental evidence that females modified investment in signalling when they perceived mating chances to be low and the oviposition date came closer. A previous behavioural study showed that old virgin *A. bruennichi* females attract more males in the field [43], which we can now attribute to males responding to increased pheromone release of older females. Taken together, these findings show that female *A. bruennichi* use pheromone signals strategically.

Several studies in other spiders found matching signalling patterns, i.e. reported a male preference for unmated over mated females and for older over younger virgin females [23,27–32]. For example, in the wolf spider *Schizocosa malitiosa*, Baruffaldi and colleagues showed that a putative pheromone is more abundant in mass spectra obtained from silk samples of older females than in those obtained from freshly moulted ones [24], and that silk from older females elicited stronger male courtship response than silk from younger ones [29]. Similar results have been obtained for the congener *Schizocosa ocreata* [27], as well as the funnel-web spider, *Agelenopsis aperta* [51], and the brown widow spider, *Latrodectus geometricus* [52]. In all species, older females were more attractive than young ones in behavioural tests. Interestingly, in *L. geometricus*, older females were less fecund [52]. The authors suggest that these females may increase pheromone production to trick males into mating with them despite their reduced fecundity. Taken together, the above studies provide suggestive evidence that females of these species adaptively modify pheromone signals, but a direct causal connection could not been shown, either because the pheromone signal was unknown, or because signalling measurements and male behaviour were not directly connected. Nevertheless, the taxonomic spread of such findings suggests that strategic use of female pheromone signals may be common among spiders.

Costs of pheromone signals can arise due to eavesdropping by predators and parasites, which has been demonstrated in some cases [53], but hardly anything is known about costs resulting from pheromone biosynthesis [12,13]. The strategic-signalling hypothesis implies that no- or low-cost signals are not expected to vary with need or with body condition, whereas costly signals should [16]. Following this logic, our findings indicate that pheromone production and/or release in *A. bruennichi* are costly. We do not know the nature and magnitude of the costs, but physiological costs appear likely for several reasons. First, the amount of pheromone per unit body mass increased in old virgins as compared with young ones. Second, the amount of pheromone released was condition-dependent. Third, young virgins also released lower absolute amounts of pheromone than old virgins, suggesting that they initially save costs. In addition, females not only start with low quantities, but also do not signal at all during the first three days after maturation ([40]; K Weiss and JM Schneider 2021, unpublished pheromone data). This is not explained by female receptivity, as many females mate during this time and even during moulting, when they are probably haphazardly found by males [54]. Finally, females ceased pheromone emission after a single copulation, which is only half the possible mating rate. One copulation is usually enough to ensure the fertilization of several consecutive egg sacs [37], but does not permit cryptic female choice [55,56]. The late start and the early shut-down might minimize possible disturbance by superfluous males, although such costs should be small and reduced by the meal that most males turn into after copulation. Alternatively or additionally, ecological costs of pheromone signals, such as eavesdropping by parasites or parasitoids, may affect the cost/benefit ratio of signalling [53]. These risks are lowered by limiting the time of signalling to a minimum, which might be the adaptive value of the latter two points. To date, we have no information regarding ecological costs in *A. bruennichi* and never observed predation in the field. However, the use of chemical cues by specialized predators on spiders has been observed [57]. In sum, the above observations strongly imply the existence of significant ecological and physiological costs of pheromone signalling for females.

The enhanced signalling effort of old virgin females and the likely occurrence of physiological costs open up another intriguing question: does the female pheromone of *A. bruennichi* constitute an honest signal (*sensu* [58]) allowing males to adaptively choose mates? Traditionally, explanations of the evolution of honest signals involve so-called ‘strategic costs' that a signaller has to pay in addition to the minimum costs needed to effectively transfer the signal (efficacy costs) [11,59]. For example, the honesty of female pheromone signals in different moths has been demonstrated to bear on physiological costs of pheromone production [12,13,60]. Honest signals may evolve without strategic costs, however, when there is no conflict of interests between sender and receiver [59,61]. Interestingly, previous studies found no or only inconsistent direct [62] or indirect [63–65] benefits of polyandry in *A. bruennichi*. Hence, females do not necessarily benefit from attracting more than one male. Males, on the other hand, have very restricted mating rates as well and should benefit from being choosy in favour of fecund females. *Argiope bruennichi* has particularly high rates of female sexual cannibalism with almost 80% of males dying during their first copulation [66,67]. Moreover, males damage their paired pedipalps during copulation [35,36], limiting them to a maximum of two copulations. Behavioural studies have shown that males adjust their mating tactic, i.e. whether they invest maximally in one female or seek to obtain a second mating with another female, according to female condition [41,42,68]. In spiders, as in other arthropods, female body mass correlates with fecundity [69] and in female *A. bruennichi*, body mass is positively correlated with age. Moreover, assuming a constant mortality risk during a female's adult life, the older a female is at the time of mating, the more likely she will survive until egg-laying [70]. Thus, the preference for older females is adaptive. Even more critical for the reproductive success of male *A. bruennichi*, however, is female mating history. Due to the high rate and effectiveness of mating plugs in this species [35,36] and the fact that males probably cannot detect plugs [63], males mating with a once-mated female have a 50% risk of fathering no offspring with this female and even if they inseminate the unplugged genital opening, they face enhanced sperm competition. Correspondingly, Schulte *et al*. [39] demonstrated that female mating status is indeed a stronger predictor of male mate choice decisions than age or condition. Interestingly, mating status had the most pronounced effect on pheromone release in our study, as once-mated females completely stopped signalling. Taken together, in *A. bruennichi*, male and female mating interests coincide with males benefiting most from choosing old virgin females, which in turn benefit most from getting mated. Thus, the strategic pheromone signal of female *A. bruennichi* may also be classified as an honest signal, even under the assumption of only low or no production costs for females.

In summary, our study complements the extensive body of behavioural evidence for pheromone-based mating decisions in spiders and experimentally demonstrates the proximate mechanism underlying the observed behaviours. More quantitative chemical studies are desirable to test the generality of our findings of strategic use of chemical signals by females. A next important step will be to identify physiological and ecological costs of pheromone production in spiders.

## Data accessibility

Datasets are provided in the Dryad Digital Repository (https://doi.org/10.5061/dryad.g79cnp5qk) [71].

The data are provided in electronic supplementary material [72].
